# A fast phenotype approach of 3D point clouds of Pinus massoniana seedlings

**DOI:** 10.3389/fpls.2023.1146490

**Published:** 2023-06-26

**Authors:** Honghao Zhou, Yang Zhou, Wei Long, Bin Wang, Zhichun Zhou, Yue Chen

**Affiliations:** ^1^ College of Electronic and Information Engineering, Zhejiang University of Science and Technology, HangZhou, ZheJiang, China; ^2^ College of Biological and Chemical Engineering, Zhejiang University of Science and Technology, HangZhou, ZheJiang, China; ^3^ Research Institute of Subtropical Forestry, Chinese Academy of Forestry, HangZhou, ZheJiang, China; ^4^ Horticulture Institute, Zhejiang Academy of Agricultural Sciences, HangZhou, ZheJiang, China

**Keywords:** 3D point cloud, phenotyping, skeletonization, Pinus massoniana seedlings, slicing

## Abstract

The phenotyping of Pinus massoniana seedlings is essential for breeding, vegetation protection, resource investigation, and so on. Few reports regarding estimating phenotypic parameters accurately in the seeding stage of Pinus massoniana plants using 3D point clouds exist. In this study, seedlings with heights of approximately 15-30 cm were taken as the research object, and an improved approach was proposed to automatically calculate five key parameters. The key procedure of our proposed method includes point cloud preprocessing, stem and leaf segmentation, and morphological trait extraction steps. In the skeletonization step, the cloud points were sliced in vertical and horizontal directions, gray value clustering was performed, the centroid of the slice was regarded as the skeleton point, and the alternative skeleton point of the main stem was determined by the DAG single source shortest path algorithm. Then, the skeleton points of the canopy in the alternative skeleton point were removed, and the skeleton point of the main stem was obtained. Last, the main stem skeleton point after linear interpolation was restored, while stem and leaf segmentation was achieved. Because of the leaf morphological characteristics of Pinus massoniana, its leaves are large and dense. Even using a high-precision industrial digital readout, it is impossible to obtain a 3D model of Pinus massoniana leaves. In this study, an improved algorithm based on density and projection is proposed to estimate the relevant parameters of Pinus massoniana leaves. Finally, five important phenotypic parameters, namely plant height, stem diameter, main stem length, regional leaf length, and total leaf number, are obtained from the skeleton and the point cloud after separation and reconstruction. The experimental results showed that there was a high correlation between the actual value from manual measurement and the predicted value from the algorithm output. The accuracies of the main stem diameter, main stem length, and leaf length were 93.5%, 95.7%, and 83.8%, respectively, which meet the requirements of real applications.

## Introduction

Pinus massoniana is the main timber tree species in southern China; its roots are rich in resin and can be used for flavor synthesis, which is widely used in the construction, papermaking, and artificial fiber industries. After the standardized cultivation of Pinus massoniana seedlings, the obtained adult plants provide high economic benefits. Pinus massoniana is one of the main tree species for afforestation in barren hills in China, and its wood is also used to make furniture and landscape decorations. At present, one of the most important problems in ecology is the local-scale coexistence mechanism driving plants of the same genus (Silvertown, 2004). By researching the phenotypic parameters of Pinus massoniana, the coexistence mechanism can be investigated, which is significant in ecological research. Simultaneously, the phenotypic morphology of Pinus massoniana seedlings can be used to predict the future growth of plants by selecting healthy seeds for cultivation. However, manually measuring phenotypic parameters is time-consuming and laborious and errors can easily occur. Three-dimensional (3D) plant models and automatic phenotypic algorithms provide an efficient and convenient method for plant structural digitization. Therefore, developing an automated method to obtain plant phenotypic parameters from 3D plant models greatly improves measurement efficiency.

In phenotypic applications, two-dimensional (2D) images or 3D point clouds are widely used to characterize the individual morphology of plants (Vázquez-Arellano et al., 2016). 2D images have been widely used in phenotypic analysis; however, due to the limitation of dimensions, 2D images cannot accurately include plant information as well as 3D data (Kaminuma et al., 2004; Gibbs et al., 2018). In particular, phenotype measurements of the leaf area, stem diameter, stem volume, and other parameters require 3D information on plants to obtain more accurate results. Usually, there are two methods of obtaining the 3D phenotypic data of plants. One method is to reconstruct 3D models by synthesizing multi-view 2D images (Park, 2005), and this method has been successfully applied to the 3D reconstruction of wheat and paddies (Pound et al., 2014). However, the limitation of this method is that the 3D reconstruction of the plant surface lacks information from texture and light changes (de Moraes Frasson and Krajewski, 2010). Another approach is to obtain the 3D modeling of plant phenotypes directly from 3D-based sensors, such as lidar, 3D laser scanners, time-of-flight (ToF) cameras, and structured light cameras. These devices have the advantages of high precision, high signal update, and strong robustness. Applications of lidar include extracting plant skeletons from images captured by lidar scanners to estimate the leaf length, leaf inclination, leaf tip length, leaf azimuth (Jimenez-Berni et al., 2018; Wu et al., 2019), leaf area index (LAI) (Zhao and Popescu, 2009), and stand volume (Dziubich et al., 2016). Laser scanners are used to estimate the leaf area, leaf angle, chlorophyll content (Eitel et al., 2010), plant height, leaf width, and main stem volume (Paulus et al., 2014), while the ToF camera is used to evaluate plant traits (Xiang et al., 2019) and classify crops (Li and Tang, 2018). Some research groups have reported that structured light cameras are also a useful tool to extract plant phenotypic data (Nguyen et al., 2015) and predict farm crop growth conditions (Rosell-Polo et al., 2015).

According to the actual environment and the phenotypic features of plants and compared with the 3D images of plants obtained by cameras based on different principles, it was found that the 3D images obtained by ToF cameras were more suitable for our study. The Microsoft Azure Kinect camera, which has high color and depth resolution and performs well in displaying 3D images of Pinus massoniana in plant phenotype applications, is a common camera that is based on the ToF principle. Teng et al. (2021) developed a 3D image acquisition system for oilseed rape plants using an Azure Kinect camera. This system collected point cloud images from six angles and obtained complete point cloud images of plants by rotating the registration. Combined with this system and the hardware facilities of the experimental environment, a set of similar image acquisition devices that obtain three-dimensional images of different plants through non-destructive methods was built.

In agricultural and forestry applications, such as plant biomass analysis, some phenotypic parameters, such as plant height, stem diameter, and leaf length, are important indicators for evaluating plant health, growth status, and effective photosynthesis ability. Because of the morphological characteristics of Pinus massoniana, its leaves grow along the main stem. To obtain the phenotypic parameters of leaves and stems, separation is very important in the whole extraction process. Some algorithms, such as the locally convex connected patches (LCCP) algorithm (Hu et al., 2020), random sample consensus (RANSAC) cylinder fitting algorithm (Fischler and Bolles, 1981), color-based region growth segmentation algorithm (Tang, 2010), curvature-based region growth segmentation algorithm (Besl and Jain, 1988), and skeletonization-based methods, are widely used in leaf and stem segmentation. Wang et al (2020). used the LCCP algorithm to segment the registered vegetable seedlings and then calculated the length, width, and surface area of segmented leaves. However, the limitation of this method is that some leaves cannot be separated from the main stem and require manual segmentation. Another segmentation method is to use RANSAC to fit the cylinder as the main stem of plants (Ghahremani et al., 2021). This method has a good effect on direct-stem plants; however, the main stem of Pinus massoniana is bent, and part of the main stem can be extracted by this method. This method also needs to manually set the RANSAC searching radius without knowing any main stem information, which is still difficult to perform. The application of curvature-based region growing segmentation is to calculate the curvature of the leaf and stem and set a threshold to separate the leaf and stem (Lin et al., 2016). This method will set different thresholds for different individuals of the same plant; thus, this method cannot be universally used. Regarding skeletonization, recent developments adopt slice clustering as the skeleton points of a plant. Then, a Hough plane (Dalitz et al., 2017) is searched according to the skeleton points, and the distance threshold to the Hough plane is used to find the main stem (Xiang et al., 2019). Another application of skeletons is to use the Laplace transform (Cao et al., 2010) to shrink the point cloud of plants to obtain the skeleton graph (Wu et al., 2019). There are also some novel algorithms, such as the raindrop algorithm, that also achieve leaf and stem separation (Zermas et al., 2020). All the above algorithms provide a reference for the phenotypic analysis of Pinus massoniana seedlings.

Since they have a special morphological structure, Pinus massoniana has significant structural differences from traditional woody plants. At present, most of the phenotypic measurements of Pinus massoniana and plants with similar structures can be made through manual estimation. In a small number of papers, the plant height, ground diameter, and crown width parameters are simply estimated using depth cameras. However, the XOY plane is used in these papers as the projection plane to calculate the stem diameter. Because the near soil part of the stem of Pinus massoniana is not parallel to the soil plane, selecting the XOY plane as the projection plane will cause a position shift of the stem on the plane and ultimately cause the low accuracy of the ground diameter, with a value of approximately 75%. Moreover, no research on the other phenotypic parameters of the leaves has been conducted in these studies. Therefore, in our research, we aim to find a more accurate method to predict these parameters and improve the prediction performance. Moreover, effectively estimating the leaf length and leaf number is proposed in our study.

In this study, based on the idea of the skeletonization of slice clustering, slices in the horizontal direction were added, and slices with skeleton points of the main stem were selected with grayscale values. Combining the idea of the projection method, the regional leaf length and leaf number of Pinus massoniana seedlings were estimated. Finally, an automated method for obtaining plant phenotypic parameters from the 3D models of plants is proposed. The overall objective of this study is to automatically extract phenotypic parameters of Pinus massoniana seedlings through 3D point cloud analysis. The specific objectives are (1) to reconstruct a 3D point cloud model of plants; (2) to develop a set of processing flows to analyze the structural characteristics of plants; and (3) to automatically extract phenotypic parameters, including plant height, stem diameter, main stem length, leaf number, and regional leaf length.

## Materials

**Figure 1:**
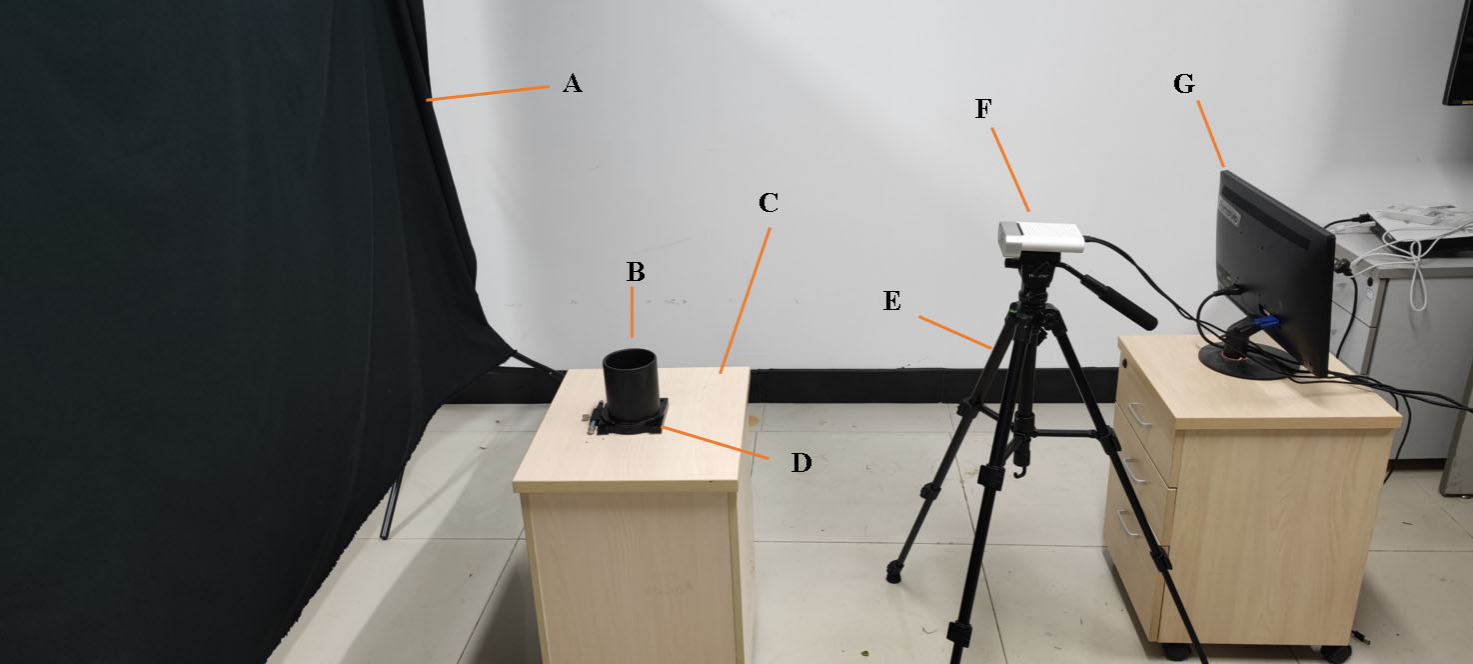
Nondestructive 3D image acquisition setup. **(A)** Curtain. **(B)** Plant placement. **(C)** Experimental platform. **(D)** Rotary table. **(E)** Yunteng691 bracket. **(F)** Aruze Kinect camera. **(G)** Computer.

A set of non-destructive 3D image collection devices, including an Azure Kinect camera, precision rotary table, bracket, black curtain, and computer, was obtained and assembled by our research group. The configuration of the computer was Intel CPU E5-2670/16G/1TB/Quadro K2000 4G DDR4, the operating system was Windows 10 Professional, and the required software was Microsoft Visual Studio 2017, OpenCV4.5.3, and PCL1.8.1. The Aruze Kinect camera integrated a color camera of 4096×3072 pixels, a depth camera of 1024×1024 pixels, and an infrared camera of 1024×1024 pixels, and it was based on the principle of ToF (L. J. T. w. p. Li, 2014). The point cloud collection device is shown in **Figure 1**. The camera position was fixed, and the Pinus massoniana seedlings were placed on the turntable, while the camera height was set to 0.5 meters, and the camera angle at the bracket was adjusted to 20 degrees down to the horizontal plane. The distance between the camera and the plant was approximately 0.4-0.5 meters. The whole plant was in the center of the camera vision view, and appropriate adjustments were made artificially according to different plants. The distance between the turntable and the curtain was 1.2-1.5 meters, and the back shadow of the plant was within the scope of the black curtain to reduce other background interference. The point cloud image was acquired every 180 degrees, and two point clouds were obtained for one plant by registration. The whole acquisition was completed by the self-developed program based on the software development kit (SDK) of the camera.

## Methods

The pipeline for phenotypic parameter acquisition involved three main steps, namely, (1) point cloud preprocessing, (2) stem and leaf segmentation, and (3) morphological trait extraction. Among them, the point cloud preprocessing step used pass-through filtering, RANSAC fitting plane and removal, radius filtering method to remove the background, experimental platform, and flowerpots. Then, the improved iterative closest point (ICP) algorithm based on feature point consistency was proposed to register the point cloud images of two angles, and complete plant point cloud images were obtained. In the stem and leaf segmentation step, the slices in the vertical direction and horizontal direction were sliced, and the skeleton points were extracted by clustering each slice. Then, the alternative skeleton points of the main stem were extracted by the directed acyclic graph (DAG) longest path algorithm, and the canopy length was estimated by local plane projection and convex hull fitting. The skeleton points of the canopy contained in the alternative skeleton points of the main stem were removed according to the canopy length, and the remaining skeleton points were regarded as points at the main stem. Finally, the skeleton points of the main stem were interpolated, and the point cloud of the main stem was obtained by k-nearest search to perform stem and leaf separation. In the morphological evaluation step, five phenotypic parameters (plant height, stem diameter, main stem length, regional leaf length, and leaf number) were estimated from the point cloud after stem and leaf separation. Our research used the Point Cloud Library (Rusu and Cousins, 2011) and OpenCV Library (Bradski and Kaehler, 2008) to implement these steps in our program.

### Point cloud preprocessing

The pipeline presented involved six steps, namely, (1) 3D point cloud generation, (2) background removal, (3) experimental platform removal, (4) discrete point removal, (5) soil and flowerpot removal, and (6) registration. In the first process, the depth image obtained by the image acquisition device was converted into 3D point cloud data. In the second step, the background curtain was removed by pass-through filtering. The third step extracted and removed the experimental platform by the RANSAC fitting plane. The fourth stage used the radius filter method to remove the discrete points in the image. The fifth stage removed the soil and the parts below the soil. Finally, the point clouds processed in the first five stages were registered by the improved ICP algorithm.

#### 3D point cloud generation

The image acquisition device obtained the depth image of the plant. For further analysis and study, the depth image was transformed into 3D point cloud data according to Equations (1) to (3) through coordinate axis transformation.


**Figure 2:**
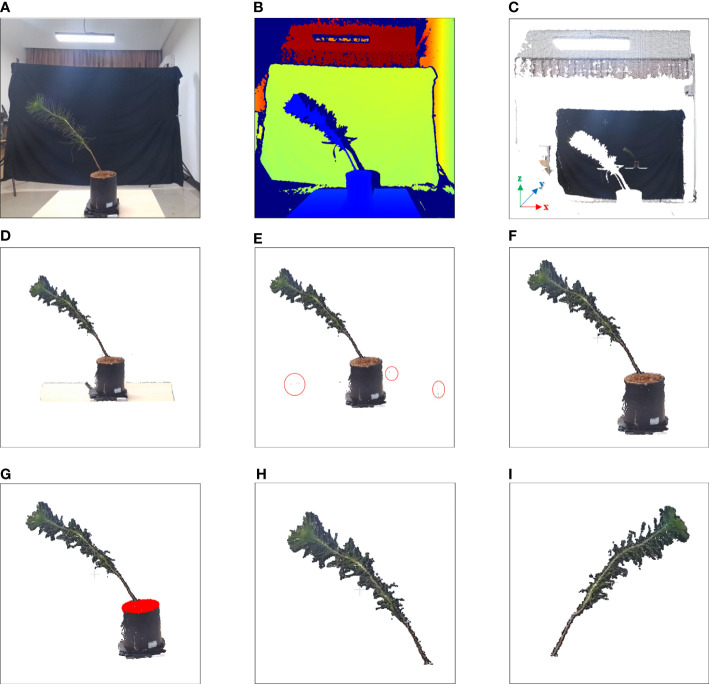
Image of point cloud preprocessing. **(A)** Real image. **(B)** Depth image. **(C)** 3D point cloud image. **(D)** Background removed point cloud image. **(E)** Experimental platform removed point cloud image; parts of discrete points are marked by red circles. **(F)** Discrete points removed point cloud image. **(G)** Soil detect image; point clouds in the soil are marked in red. **(H)** Registered point cloud image of 0 degrees. **(I)** Registered point cloud image of 180 degrees.

(1) Y = d

(2) $$X\;=\;Z\left(x-c_{x}\right)/fx$$

(3) $$Z\;=\;Z\left(y\;-\;c_{y}\right)/f_{y}$$

where x and y represent the 2D coordinates provided by the depth image according to the camera coordinate system, d represents the depth value information directly provided by the depth image, $c_{x}$ and $c_{y}$ represent the coordinates on the X-axis and Y-axis of the lens aperture center, respectively, and $f_{x}$ and $f_{y}$ are the focal lengths on the X-axis and Y-axis of the camera, respectively. The three-dimensional coordinates (X, Y, Z) of the corresponding cloud points could be obtained by combining the above formula, the X-axis represents the horizontal information of the point cloud, the Z-axis represents the height information of the point cloud, the Y-axis represents the depth information of the point cloud. The real image of the plant is shown in **Figure 2A**, and the depth image of the plant is shown in **Figure 2B**. The point cloud image of the plant is shown in **Figure 2C**.

#### Background removal

The main object was the plant, but the point cloud data generated contained some background objects, such as background curtains, loading platforms, and flowerpots. These background objects were not the targets to be measured and interfered with the point cloud analysis of subsequent plants. The background was removed by pass-through filtering. The upper and lower boundaries of the pass-through filtering were obtained using Equations (4) and (5):


(4) $$PT_{lb\;}\;=\;maxPt.z$$

(5) $$PT_{ub\;}\;=\;\left(d_{cc}\;-\;d_{cp}\right)/d_{cc}*maxPt.z$$

where $PT_{lb\;}$ and $PT_{ub\;}$ represent the near-far boundary in the depth direction of the direct filtering, as well as the distance between the lens to the curtain and the distance between the loading platform to the curtain, respectively. $d_{cc}$ represents the distance between the camera and the experimental curtain, $d_{cp}$ represents the distance between the camera and the plant, and maxPt.z represents the maximum value of the depth direction of all points in the point cloud. Hence, $PT_{lb\;}$ and $PT_{ub\;}$ were two boundaries, and the point clouds outside of the boundaries were removed. Data obtained from the above two formulas were used as the input parameters to perform direct filtering to remove the background. The background removed point cloud image is shown in **Figure 2D**.

#### Experimental platform removal

The loading platform, which is considered an irrelevant variable, had no effect on the measurement process and interfered with the point cloud analysis of subsequent plants. The point cloud of the experimental platform was detected by RANSAC plane fitting (Fischler and Bolles, 1981). The experimental platform removed point cloud was obtained by subtracting the background removed point cloud from the experimental platform point cloud. The experimental platform removed point cloud image is shown in **Figure 2E**.

#### Discrete point removal

Due to the limitations of the camera accuracy and the RANSAC plane fitting algorithm, the point cloud without the background and loading platform objects contained some sparse noise points, which had low point density. A radius-based outlier filter was used to remove these discrete noise points. The setting parameters of the radius filter were a custom search radius r and the number of adjacent points k in the search radius r . If the number of points in the search radius was less than the specified value k , the point was removed as a noise point (Dziubich et al., 2016). In this study, r = 0.01m and K = 10 were tested to effectively reduce the number of noise points without damaging the original point cloud image structure. The point cloud after removing discrete points by radius filtering is shown in **Figure 2F**.

#### Soil and flowerpot removal

In the next registration step, the method of feature point consistency was used; however, the flowerpots interfered with the registration step, and it was necessary to remove the soil of the plant and the flowerpots below the soil. The height of the flowerpots was fixed at 0.12 m in our experiment, and 0.07 m to 0.12 m above the experimental platform was selected as the soil area for pass-through filtering. The points above the soil, which are regarded as the point cloud area after the soil and flowerpot were removed, were the inputs of the registration step, as shown in **Figure 2G**.

#### Registration

This step registered point clouds collected from two angles after the preprocessing step. The registration step processed coarse registration first and then fine registration. Two preprocessed point clouds, the source point cloud, and the target point cloud, were the input of the registration step. In the coarse registration process, the KD-tree search method was used to find k adjacent points at a point in the point cloud, and the k points were fitted to the minimum quadratic plane, where the k value was set to 10. Principal component analysis (PCA) (Feng et al., 2014) was applied to the cloud points of the quadratic plane, and the normal vector corresponding to the nonzero minimum eigenvalue was considered the local normal vector. The above steps were looped until all points of the source and target point clouds were traversed. After the normal vectors of all points were obtained, the angle of normal vectors between the self-point and its adjacent points was calculated, and the mean value of these angles was obtained at the same time. The appropriate angle mean threshold was set, and if the angle mean was greater than the threshold, the point was used as the feature point in the source point cloud and the target point cloud. The fast point feature histogram (FPFH) descriptors of the feature point cloud were calculated, and the coarse registration of the target point cloud was carried out using the sampling consistency method (SAC-IA) (Rusu et al., 2009).

Improved ICP registration based on the KD-tree adjacent search algorithm was used for fine registration of the target point cloud after coarse registration (Zhang, 1994). Finally, the target point cloud and source point cloud after registration and rotation were combined into a complete point cloud image. The registered point clouds of 0 degrees and 180 degrees are shown in **Figures 2H, I**, respectively.

### Stem and leaf segmentation

The pipeline presented involved four steps, namely, (1) skeletonization, (2) main stem alternative skeleton point extraction, (3) main stem skeleton point extraction, and (4) main stem cloud point restoration. In the first stage, the preprocessed point cloud was sliced, clustering was performed on each slice, and the slice of the stem was selected by grayscale values. In the second stage, the minimum spanning tree (MST) was established according to the skeleton points, and the alternative skeleton points of the main stem were extracted by the DAG longest path algorithm. In the third stage, the length of the plant canopy was estimated by the local projection method. According to the length of the canopy, the canopy skeleton points contained in the alternative skeleton points of the main stem were removed, and the skeleton points of the main stem were obtained. Finally, the skeleton points of the main stem were interpolated, and the point cloud of the main stem was obtained by k-nearest search searching. The remaining point cloud was regarded as the area in which the leaf part was located to perform stem and leaf segmentation.

#### Skeletonization

An improved skeletonization method based on the phenotypic characteristics of Pinus massoniana seedlings was proposed. First, the slice along the Z-axis of the point cloud was performed, and a total of 30 slice layers were obtained. Then, Euclidean clustering was applied to the point clouds of each slice layer, and the corresponding clusters in each slice layer were obtained (Rusu and Cousins, 2011). However, due to the structural characteristics of Pinus massoniana, the clusters of Z-axis slices involved leaves and main stem parts. It was difficult to distinguish whether the cluster was from the leaf part or the main stem part, while the centroid was extracted from the different clusters of the slice layer. Therefore, further processing was needed at the slice layer along the X-axis.

**Figure 3:**
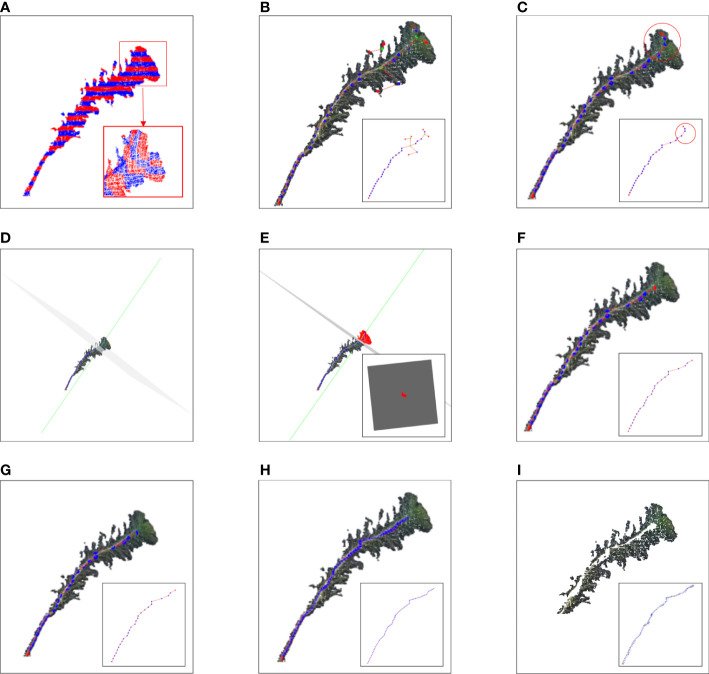
Image of the stem and leaf segmentation. **(A)** Point cloud slices along the Z-axis and X-axis with adjacent slice layers distinguished by different colors. **(B)** Diagram of the relationship between the skeleton points. The green point, red point, and blue point represent the junction, vertex, and internal node, respectively. **(C)** Skeleton points of the main stem with the canopy; the red circle represents the canopy. **(D)** The green line represents a normal composed of a fifth skeleton point and the adjacent skeleton points above it. The gray normal represents a projection plane perpendicular to the normal. **(E)** The red part represents the projection of the top canopy on the tangent plane. **(F)** Skeleton point of the main stem after removing the canopy. **(G)** Main stem skeleton points before interpolation. **(H)** Main stem skeleton points after interpolation. **(I)** Point cloud after removing the main stem.

**Figure 4:**
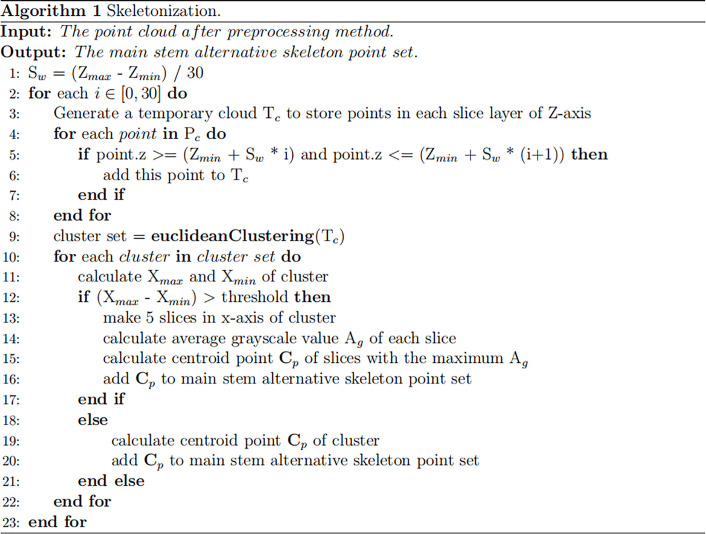
Algorithm for skeletonization.

The maximum differential value of the X-axis coordinates of the point cloud along the Z-axis of each slice layer in the cluster was calculated, and the corresponding cluster along the Z-axis slice layer, whose differential value was greater than the threshold, was sliced twice along the X-axis direction (the number of slices along the X-axis was 5). The slicing point cloud image along the Z-axis and its horizontal direction subdivision for each Z-axis slice layer is shown in **Figure 3A**. The average grayscale values of all slice layers in the same cluster along the X-axis were calculated. The slice layer with the largest average grayscale value of five horizontal slice layers was taken as the main stem location. The centroid of this X-slice layer was calculated and used as the skeleton alternative point of the corresponding cluster for the main stem of Pinus massoniana on this Z-axis slice. If the maximum differential value of the X-axis coordinates was less than the threshold, the centroid of the cluster was directly calculated and used as the skeleton alternative point of the cluster corresponding to the main stem of Pinus massoniana on this Z-axis slice. According to the above steps, traversing all slice layers along the Z-axis, the skeleton alternative points of the main stem were collected. The algorithm for skeletonization is shown in **Figure 4**.

**Table:** 

Nomenclature in Algorithm1	
*Z_max_ Z_min_*	Maximum and minimum of the Z-axis values in the preprocessed point cloud
*X_max_ X_min_*	Maximum and minimum of the X-axis values in the point cloud
*P_c_*	Preprocessed point cloud
*S_w_*	Slice layer width


#### Main stem alternative skeleton point extraction

The alternative skeleton point clouds obtained after the skeletonization step were discrete, and it was necessary to establish adjacent relationships between the skeleton points. The MST was used to establish this relationship for alternative skeleton point clouds, and the Kruskal algorithm (Kruskal, 1956) was used to build the MST of all alternative skeleton point sets of the main stem. By searching up from the root node of the MST, the directed acyclic graph between the points was generated according to the adjacent relation formed by the MST. The diagram of the relationship between the skeleton points is shown in **Figure 3B**. Then, the DAG longest path algorithm was used to search the longest path from the root node in a directed acyclic graph (Healy and Nikolov, 2001). The skeleton points in the longest path were set as the skeleton points of the main stem with canopy, which is shown in **Figure 3C**.

#### Main stem skeleton point extraction

As shown in **Figure 3C**, the top of Pinus massoniana is a canopy, and it did not belong to the main stem part. As a result, it was necessary to remove the canopy part involved in the skeleton points of the main stem with the canopy. Therefore, according to the distance information between the skeleton points provided by the MST, the canopy length was estimated, and the skeleton points in the canopy range were removed from the top based on the estimated length. Thus, the skeleton points of the main stem and the canopy were separated.

**Figure 5:**
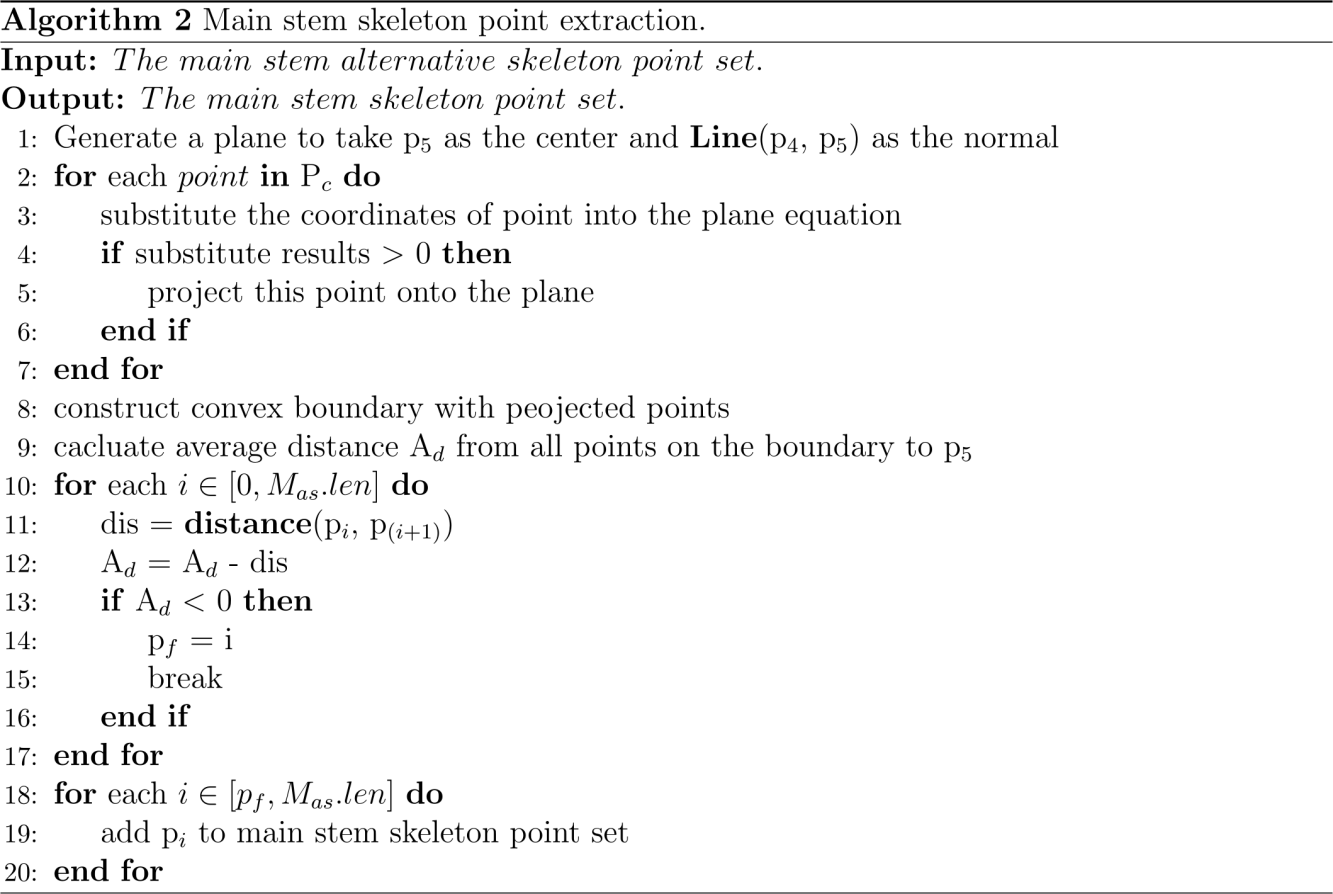
Algorithm for main stem skeleton point extraction.

In this study, the number of point clouds of the main stem skeleton with a canopy was investigated. It was found that the boundary point between the canopy and the main stem was near the fourth or fifth point of the skeleton points with the canopy from top to bottom in our samples. Therefore, the fifth point was selected as the center of the following local projection method. The highest point of the point cloud in the main stem skeleton without the canopy was the starting point, and the MST was traversed down to the position of the fifth skeleton point. Then, the plane was formed under the centroid of the fifth skeleton point and the skeleton point above it, which was perpendicular to the normal. The normal and projection planes are shown in **Figure 3D**. The point cloud above the plane was projected onto the plane. Points projected onto the plane are shown in **Figure 3E**. The convex boundary of the 2D projection point cloud was extracted based on the convex hull algorithm (Cupec et al., 2020). The average distance from all points on the boundary to the search center (fifth point) was determined to be the threshold of the specified canopy length. From the top of the skeleton point set of the main stem with the canopy, the sum of the paths between the skeleton points according to the distance relationship provided by the MST was calculated. During searching, if the sum of the paths was greater than the threshold, the search was stopped. The skeleton point from this point to all the collections from the top was discarded, and the remaining skeleton points were the skeleton points of the main stem. The skeleton point of the main stem is shown in **Figure 3F**. The algorithm for main stem skeleton point extraction is shown in **Figure 5**.

**Table:** 

Nomenclature in Algorithm2	
*p_i_*	The i-th point of the alternative main stem skeleton point from top to bottom
*Line*()	The line between two points
*P_c_*	Preprocessed point cloud
*M_as_*	Main stem alternative skeleton point set
*M_as_*.*len*	The length of the main stem alternative skeleton point set
*p_f_*	Dividing point of the canopy and main stem part


#### Main stem point cloud restoration

The purpose of this step was to restore the main stem according to the skeleton point cloud of the main stem. The k-nearest search method was used to search the point cloud along the skeleton points of the main stem within the set radius range, and if the search radius was set too large, the point cloud of the leaf part might be wrongly restored to the main stem. The distance between the main stem skeleton points was uniform, and the large distance caused leaf point cloud loss in the search. Therefore, in this study, the main stem skeleton points were interpolated to solve the above problem.

**Figure 6:**
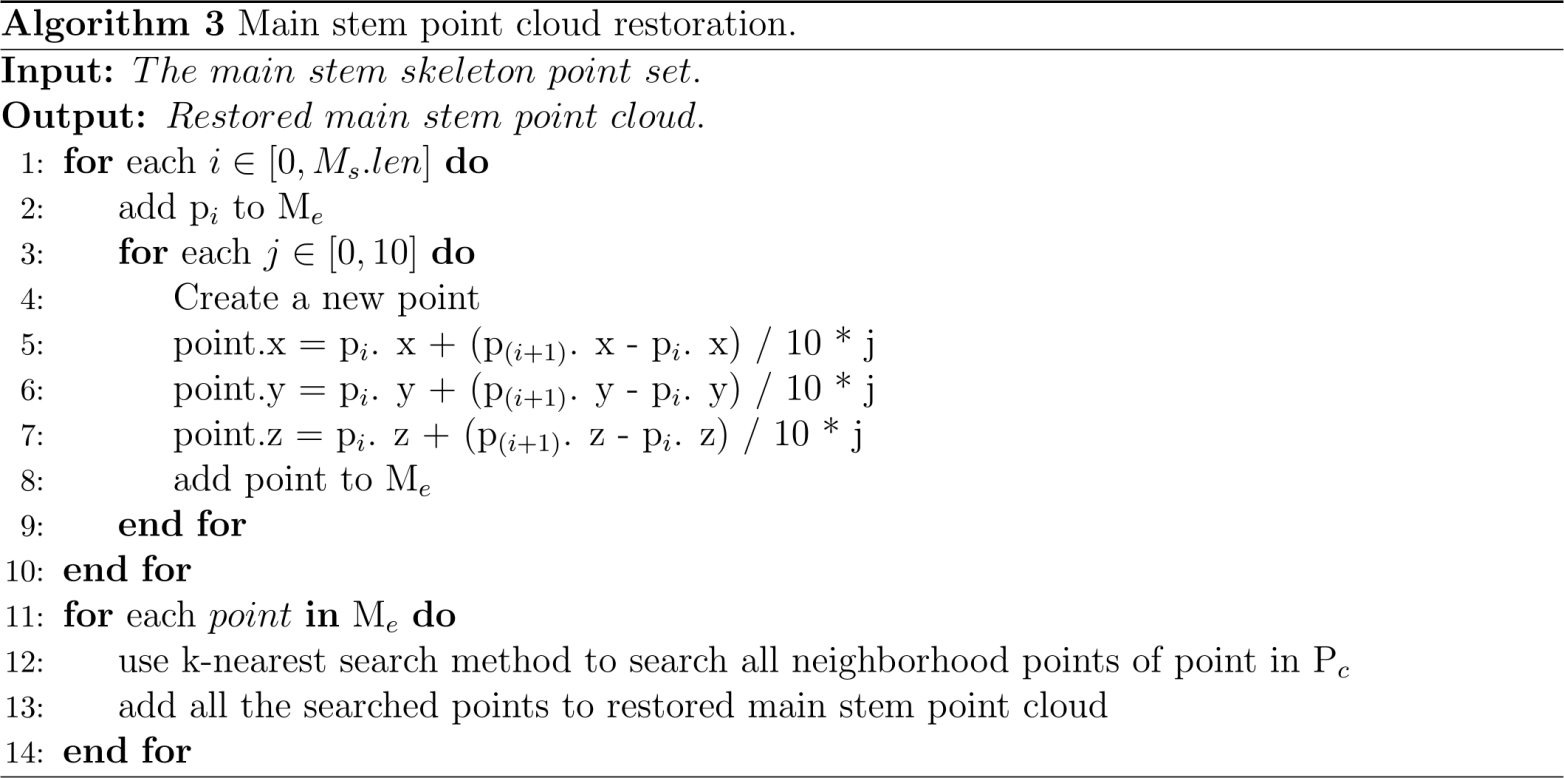
Algorithm for main stem point cloud restoration.

Because the near-soil parts of the main stem of Pinus massoniana had no leaves, the point cloud of the main stem extracted by the k-nearest search method did not involve the leaf point cloud, and the diameter of the main stem in this region was calculated using the algorithm mentioned in the morphological evaluation step and used as an interpolation step length. The number of new skeleton points inserted between the two skeleton points was determined from the distance between the two skeleton points divided by the interpolation step length, then linear interpolation was carried out. All the adjacent skeleton points were traversed to complete the interpolation expansion of the whole skeleton point set. The main stem skeleton points before and after interpolation are shown in **Figures 3G, H**. For the expanded main stem skeleton points after interpolation, a k-nearest search was used to search the points within the radius (the search radius was the main stem diameter), and all the searched points could be regarded as the main stem part. When the preprocessed point cloud was subtracted from the point cloud of the main stem, the point cloud of leaves was obtained, and the stem and leaf segmentation step was realized. Point clouds that did not contain the main stem are shown in **Figure 3I**. The algorithm for main stem point cloud restoration is shown in **Figure 6**.

**Table:** 

Nomenclature in Algorithm3	
*p_i_*	The i-th point of the main stem skeleton point from top to bottom
*P_c_*	Preprocessed point cloud
*M_s_*	Main stem skeleton point set
*M_s_*.*len*	The length of the main stem skeleton point set
*M_e_*	Expanded main stem skeleton point set


### Morphological trait extraction

Five phenotypic parameters, including the plant height, stem diameter, main stem length, regional leaf length, and leaf number, were calculated automatically using the point cloud in the output of the stem and leaf segmentation step. The stem diameter was calculated using the local projection method. Part of the main stem was projected onto the tangent plane formed along the skeleton point above the soil. The stem diameter was represented by the short axis length of the ellipse by 2D ellipse fitting. The main stem length was obtained by calculating the distance between adjacent skeleton points. For the regional leaf length, we took a region along the skeleton point of the main stem every 5 cm, and the point cloud in the region was projected to the local tangent plane. The convex hull method was used to estimate the length of the projection boundary to the center, and the estimated length was used as the local leaf length. The number of leaves can also be determined by searching along the main stem skeleton point, and every 5 cm was regarded as a region. The distance density of this region was calculated, and the number of leaves in this region was estimated by the linear relationship between the distance density and leaves. Then, the total number of leaves was estimated from the density.

#### Plant height

The plant height was defined as the distance between the soil plane and the top of the canopy, which is a useful and frequently measured trait in agronomic research (Moles et al., 2009). Using the highest point of the whole plant as the top of the plant, the plant height was calculated using **Eq. 6**:


(6) $$H\;=\;Z_{max}\;-\;Z_{soil}$$

where $Z_{max}$ and $Z_{soil}$ represent the Z-axis of the highest point of the point cloud after preprocessing and the Z-axis of the soil plane, respectively.

#### Stem diameter

The measurement position of the stem diameter was close to the soil, and the measurement value of this position was for the whole stem. The routine method selected the part 2-5 cm above the soil and sliced along the Z-axis with a thickness of 1 cm. The point cloud of each selected slice layer was projected onto the XOY plane, a 2D ellipse was fitted using these 2D projected points, and the minor axis of the ellipse was taken as the estimated value of the stem diameter. However, the slice layer of the plant along the Z-axis was not perpendicular to the XOY plane, leading to a certain deviation of the points on the XOY plane. Therefore, we proposed an improved algorithm for skeletonization and projection and for calculating the stem diameter.

**Figure 7:**
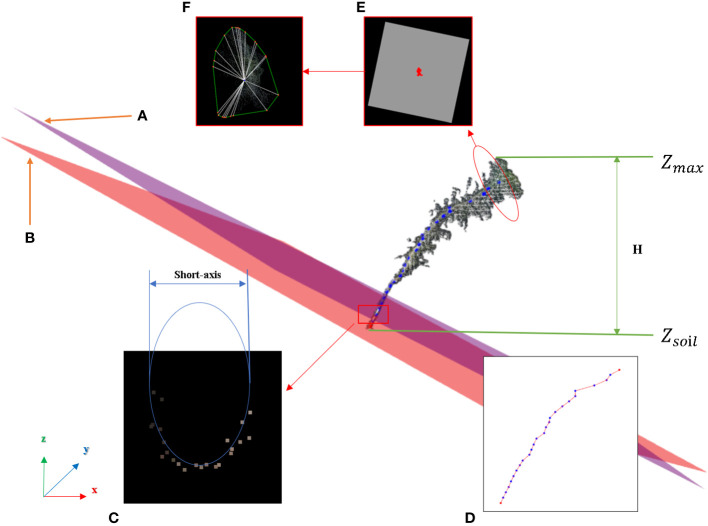
Image of the morphological traits extraction. **(A)** Upper plane. **(B)** Lower plane. **(C)** 2D projection of the main stem point cloud, short-axis of the ellipse as the stem diameter. **(D)** Main stem skeleton points for calculating the main stem length. **(E)** 2D points projected onto the lower plane. **(F)** Convex boundaries and links between the boundaries and center point.

The expanded skeleton points of the main stem were used, and all skeleton points in the 2-4 cm region above the lowest skeleton point were selected. At the initial stage, the normal was formed by the skeleton point at the lowest position. Then, the plane that was perpendicular to the normal was generated while the lowest skeleton point was the center, and the generated plane was denoted as the lower plane. The skeleton point above the lowest skeleton point was taken as another center, and the normal was generated with the skeleton point above another center. Another plane perpendicular to the normal was generated and was denoted as the upper plane. The upper and lower planes are shown in **Figures 7A, B**, respectively. The points between the upper plane and the lower plane were projected to the lower plane after the preprocessing step. Every 1 cm was sliced as a layer. Then, the 2D ellipse fitting operation was performed on these projection points, the short axis length of the fitted ellipse was used as the diameter, and the average value of the diameters was the stem diameter of the main stem. The projection of points is shown in **Figure 7C**.

#### Main stem length

The stem is an important organ that connects the transport path; Pinus massoniana seedlings have only one main stem, and leaves grow around the main stem (Zhuo et al., 2010). The main stem skeleton point cloud is shown in **Figure 7D**. By traversing from the lowest point of the skeleton to the highest point, the distance between the adjacent points in the MST was determined, and the sum of all the distances was the main stem length.

#### Regional leaf length

Since the growth conditions of different parts of Pinus massoniana were different, the average leaf length of different parts was estimated in this study. The region of the skeleton point cloud of the main stem was taken every 5 cm along the main stem direction. The lowest position of the skeleton points in the region and its adjacent skeleton points were selected and used to form one normal vector. Then, the plane that was perpendicular to the normal vector was generated where the lowest position skeleton point was the center and was denoted as the lower plane. The highest skeleton point of the region was the center, with its adjacent skeleton points below the form of a normal vector, generating a plane perpendicular to the normal that is denoted as the upper plane. The points between the upper plane and the lower plane were vertically projected to the lower plane, as shown in **Figure 7E**. The convex boundary of the 2D projection point cloud was extracted by the convex hull algorithm (Cupec et al., 2020), which is shown in **Figure 7F**.

The average distance from all points on the boundary to the center of the lower plane was determined, and the calculated stem diameter was the threshold. If the average distance was greater than the threshold, the average distance was used for the average leaf length of the region. If it was less than or equal to the threshold, it was considered that there were no leaves in this region. The above algorithm could be used to estimate the average leaf length of the parts below the canopy of Pinus massoniana along every 5 cm interval of the main stem.

#### Leaf number

The Pinus massoniana leaves were dense and large in number. It was difficult to obtain separate leaf images even using a high-precision scanner. The point cloud distance density is an important analysis index and can be used to analyze plant phenotypic characteristics. For example, by using the different densities of the point clouds of stems and leaves in the horizontal direction, the support vector machine can be applied to classify the stems and leaves by density (Liu et al., 2020). In our study, the same regional segmentation method as the regional leaf length step was used to calculate the average distance density of the point cloud in different segmentation regions, and the number of leaves in corresponding regions was counted. It was found that the distance density of the point cloud had a certain linear relationship with the number of regional leaves. Based on this relationship, we proposed a plant leaf estimation algorithm based on the distance density method (C.-H. Lin et al., 2018). Moreover, the average distance density of the point cloud can be expressed by **Eq. 7** and **Eq. 8**:


(7) $$d_{p\;}=min\left(dis\left(p,q\right)\right),q=1\cdots N,p\neq q$$

(8) $$\bar{d}\;=\;\frac{1}{N}\sum_{p=1}^{N}d_{p}$$

dis(p,q) represents the Euclidean distance between Point *p* and any other point q in a point cloud with N point numbers, and $d_{p}$ is the minimal value of all the distances. The smaller $\bar{d}$ is, the sparser the point cloud distribution; the larger $\bar{d}$ is, the denser the point cloud distribution. Based on the above formula, the algorithm for calculating the number of each leaf was as follows. The skeleton point cloud region of the main stem was used for estimating every 5 cm along the main stem, and the lowest position of the skeleton points in the region was selected as the center. With one upper adjacent skeleton point, a normal vector can be formed, and the plane that was perpendicular to the normal vector was generated and denoted as the lower plane. If there were leaves in this region (the determination method was the same as the determination method in the local leaf length step), the average distance density between these points can be calculated. Through the linear relationship between the average distance density and the number of regional leaves, the number of leaves in this region can be obtained. The leaf number of each region was calculated, and the total leaf number of Pinus massoniana was obtained by the summation of all the regions.

## Results

**Figure 8:**
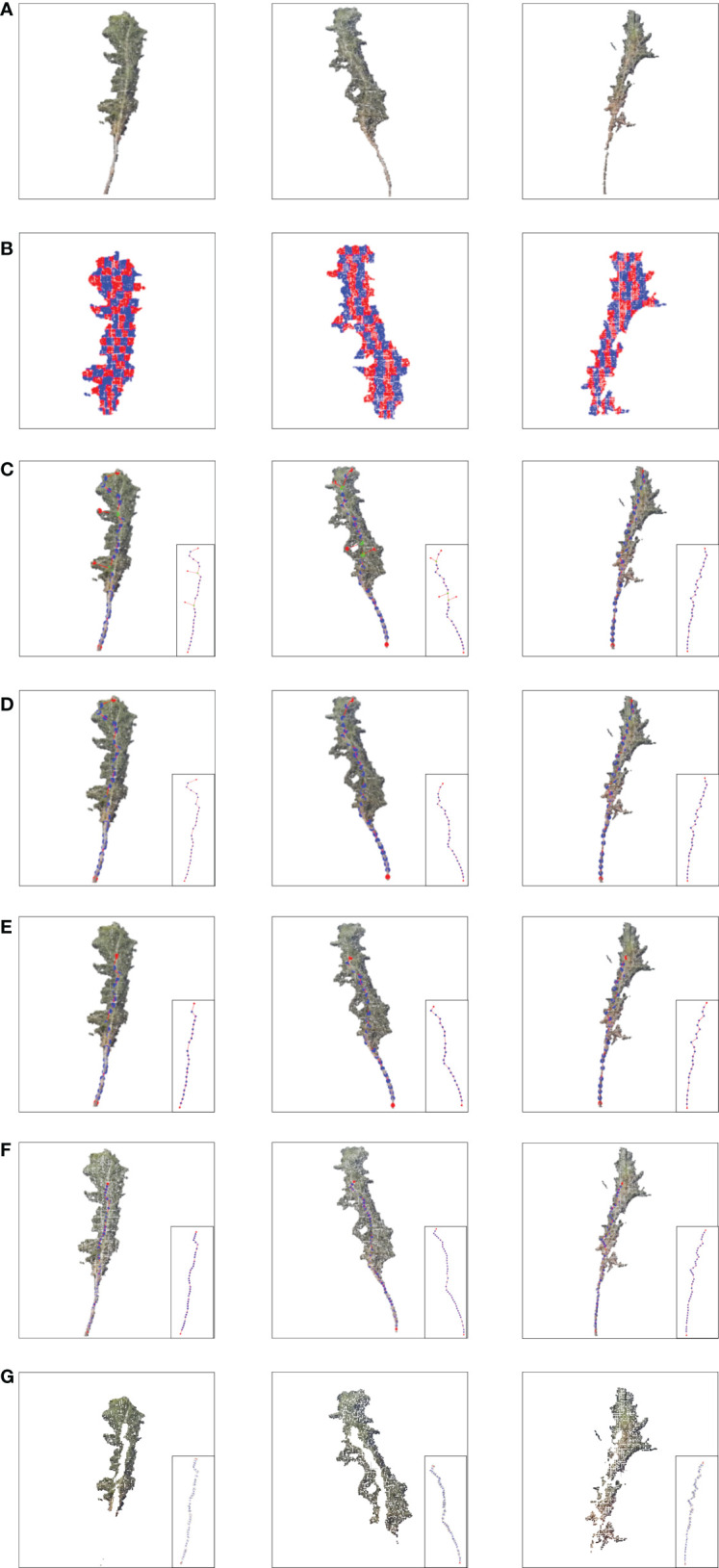
Stem and leaf segmentation procedure visualization of different heights. **(A)** Input cloud point. **(B)** Slice in the X-axis and Z-axis. **(C)** Extracting alternative skeleton points of the main stem according to the centroid and generating MST according to the skeleton points. **(D)** DAG longest path algorithm searches for the skeleton points of the main stem with a canopy. **(E)** Main stem skeleton points after removing the canopy. **(F)** Main stem skeleton points after interpolation expansion. **(G)** After removing the main stem point cloud, the plant point cloud only contains leaves.

**Figure 8** shows the stem and leaf segmentation results of three representative Pinus massoniana plants with height ranges of 25-30 cm, 20-25 cm, and 15-20 cm. The three-dimensional surface visualization found that the 3D model was close to the real sample, and the skeleton of the main stem from the point cloud model is mostly consistent with the skeleton of the actual plant. We can note that the skeleton points of the third plant in **Figure 8** have a small deviation from the real stem because this deviation has many leaves and shelters the main stem. Thus, the skeleton points of this part that were found by the grayscale value clustering deviate; however, no more than 5% of the skeleton points had this deviation, and the actual impact was not significant. Overall, the results show that the stem and leaf segmentation algorithm could effectively separate the main stem and leaf from the 3D point cloud of Pinus massoniana plants.

### Accuracy assessment

In this study, 100 Pinus massoniana seedlings at 15–30 cm were collected as part of the experiment. The accuracy of the algorithm was evaluated by the correlation coefficient (R), root mean square error (RMSE), and mean absolute error (MAE). RMSE and MAE were defined as **Eq. 9** and **Eq. 10**.


(9) $$RMSE=\;\sqrt{mean{\left(est\;-\;act\right)}^{2}}\;$$

(10) MAE = mean(|est − act|)

where est and act denote the estimated values and actual values from manual measurements, respectively.

**Figure 9:**
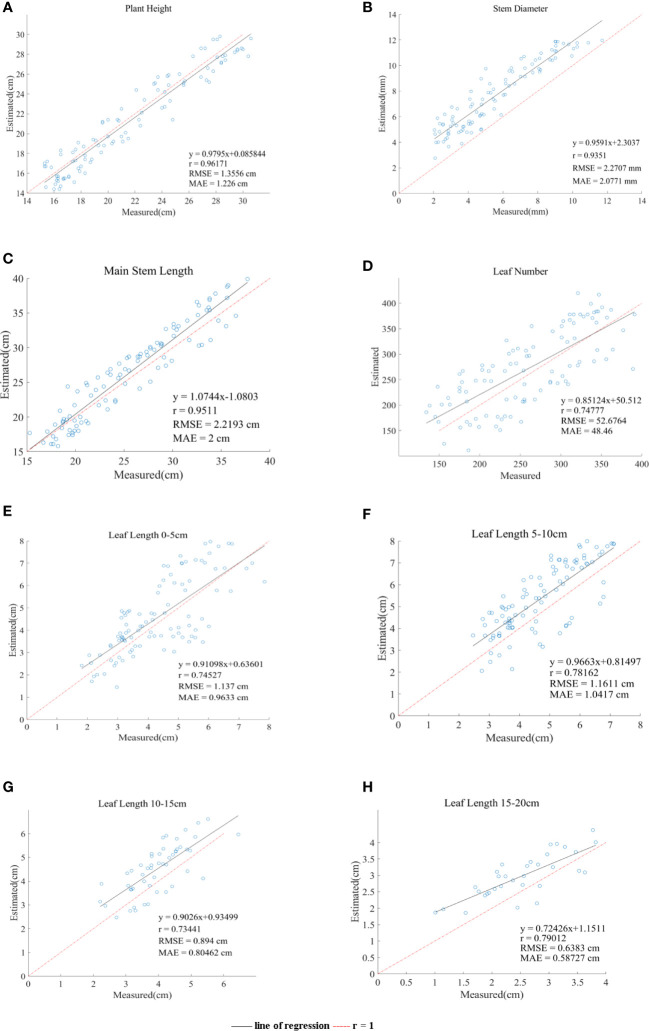
Comparison of the six phenotypic parameters system estimates and manual measurements. (**A–H**) line of regression represents the straight line fitted by the estimated value. r = 1 represents estimated value is equal to actual value and regards as a reference line.

A comparison of the estimates and manual measurements of the phenotypic parameters is shown in **Figure 9**. The results showed that this set of algorithms was suitable for the phenotypic parameter extraction of Pinus massoniana seedlings, especially for plant heights and main stem lengths. Among them, the R between the estimated plant height and the artificially measured value was 0.96, and the RMSE and MAE were 1.35 cm and 1.21 cm, respectively; the R between the estimated plant height and the artificially measured value was 0.93, and the RMSE and MAE were 2.27 mm and 2.07 mm, respectively; the R between the estimated main stem length and the artificially measured value was 0.95, and the RMSE and MAE were 2.21 cm and 2 cm, respectively; the R between the estimated leaf numbers and the artificially measured value was 0.75, and the RMSE and MAE were 52 cm and 48 cm, respectively; the R between the average leaf length 0-5 cm below the canopy and the measured values was 0.74, and the RMSE and MAE were 1.13 cm and 0.96 cm, respectively; the R between the average leaf length 5-10 cm below the canopy and the measured values was 0.78, and the RMSE and MAE were 1.16 cm and 1.04 cm, respectively; the R between the average leaf length 10-15 cm below the canopy and the measured values was 0.73, and the RMSE and MAE were 0.89 cm and 0.8 cm, respectively; the R between the average leaf length 15-20 cm below the canopy and the measured values was 0.79, and the RMSE and MAE were 0.63 cm and 0.58 cm, respectively.

Regarding the plant height, the regression line of the data points was in good agreement with the reference line of the diagonal (R = 1), as shown in **Figure 9A**, which verified that the algorithm was suitable for measuring the plant height of Pinus massoniana. The average height of all artificially measured plants was 21.75 cm, and the average absolute error was 5.5% of the average. The reason for this error was that the soil plane was slightly uneven, resulting in the overestimation or underestimation of the lowest soil plane. However, this error can be ignored.

Regarding the stem diameter, **Figure 9B** shows that the estimated value of the system is generally greater than the measured value, which was caused by the ToF principle of the Aruze Kinect camera. Moreover, the reflection of the edge leads to the expansion of the point cloud around the stem.

Regarding the stem diameter calculation step, we have shown that the average absolute error of the stem diameter calculated by selecting the tangent plane of the local skeleton point of the main stem as the projection plane was 26.6% of the manual measurement average, while the average absolute error of stem diameter calculated using the XOY plane as the projection plane was 42.2% of the manual measurement average. Therefore, using the projection plane used in this study to calculate stem diameter greatly improves the accuracy.

Regarding the length of the main stem, **Figure 9C** shows that the correlation between the estimates and manual measurements is close to 1. The average length of the main stem of all plants measured manually was 25.6 cm, and the average absolute error was 7.8% of the average. It was proven that this algorithm is applicable to the length of the main stem. The random error may be caused by the following two reasons. First, the main stem might be partially blocked by the leaves, resulting in a deviation between the slice layer of the main stem selected by the grayscale value and the slice layer of the actual main stem. Second, the grayscale of withered Pinus massoniana leaves was similar to that of the main stem, thus interfering with the system. However, the object of this study was to analyze the seedling stage, with few or no withered leaves, and this impact factor can be ignored.

Regarding the leaf number, we proposed the density method to estimate the leaf number of Pinus massoniana. The regional leaf number and density of Pinus massoniana seedlings were calculated, and the linear relationship between the leaf number and density was obtained using the least square method. The relationship between the estimates and manual measurements is shown in **Figure 9D**. Because of the least square method (linear relationship), the number of leaves deviated from the actual value for the nonlinear relationship. The average number of leaves measured by hand was 259, and the average absolute error was 18.5% of the average measured by hand. It was proven that this estimation method had certain feasibility. This method could avoid the high cost caused by using a high-precision scanner and reduce the time consumed by manual measurement.

Regarding the regional leaf length, **Figure 9E–H** shows the average leaf length of 0-5 cm, 5-10 cm, 10-15 cm, and 15-20 cm below the canopy. The average absolute errors were 22.2%, 22.3%, 20.1%, and 23% of the average manually measured values, respectively. The main reason for these errors was that there are few long leaves, and these leaves would be included by the convex hull method, causing corresponding errors. In general, the estimated leaf length of the system was close to the actual leaf length, which also proved that the algorithm in this study was feasible.

We also used a box plot for analysis. However, in the measurement of each phenotypic parameter, there were mostly no discrete points with values that were too large or too small. The line chart could show all the information of the box plot and showed some information that the box plot did not have. Therefore, in this paper, we used a line chart for analysis.

## Discussion

In this study, a set of automated methods that measure the phenotypic parameters of Pinus Massoniana seedlings was developed to process images collected by a 3D image acquisition device through non-destructive means. The five parameters of interest were plant height, stem diameter, main stem length, regional leaf length, and leaf number, and they were automatically obtained from the original image. Because of the complex phenotypic characteristics of Pinus massoniana, there have been few studies on such plants. Our study focuses on the stem-leaf separation steps and phenotypic parameter extraction.

As an important step, stem and leaf segmentation could provide great convenience for the extraction of phenotypic parameters of subsequent plants. We also used three kinds of skeletonization methods to analyze Pinus massoniana seedlings based on skeleton contraction, local feature, and slicing. One of the typical skeleton contractions was Laplace skeletonization, and the core idea of Laplace skeletonization was to search the plant point cloud in the stem direction through matrix transformation, which had a good effect on gramineous plants, but it is not applicable for curved main stem. The second kind of skeletonization method was based on feature; however, the leaf part and stem part of Pinus massoniana were overlapping, and it was difficult to find the proper feature. The third type was based on slicing. However, the main stem of Pinus massoniana was curved, and some of the skeleton points of the main stem were out of the range of the extracted Hough plane, resulting in some main stem skeleton points being missed (comparative tests are included in the **Supplementary Materials**).

However, the idea of slicing provided much inspiration for our skeletonization, for Pinus massoniana, the Z-axis slice layer contained both the stem part point cloud and the leaf part point cloud. Then, slice subdivision was performed along the X-axis. We compared the features of each slice layer and found the characteristics of the slice layer where the main stem was located.

Regarding the phenotypic parameters, the accuracy of the plant height, stem diameter, and main stem length reached 96.3%, 84.9%, and 95.7%, respectively. Regarding the leaf length, the leaves in different regions of this study were analyzed. The growth direction of Pinus massoniana leaves was mostly perpendicular to the main stem, so the projection method was used to project the 3D point cloud to 2D for calculation to reduce the complexity of the calculation.

Although we could not obtain the accurate regional leaf length, the estimation accuracy was 83.8%, which proved that our estimation method still had feasibility. Regarding the leaf number, by comparing the local density and local leaf number of multiple groups of samples, it was found that there was a certain linear relationship between the density and leaf number. Thus, an algorithm based on distance density was designed to estimate the leaf number. Overall, there would be approximately a 15% error, but the Pinus massoniana seedling itself had hundreds of leaves; even if artificially counted, there was still approximately a 10% error. Thus, this range of error was reasonable.

## Conclusion

In this study, a low-cost 3D phenotypic system based on the Azure Kinect camera was built, and an automatic measurement method for five phenotypic parameters of Pinus massoniana with a height range of 15–30 cm was proposed. The experimental results in this study provide an efficient and economical solution for plant phenotypic feature extraction, which could promote genome research and plant breeding programs.

Our future work will focus on applying this algorithm to a three-dimensional imaging platform developed by our group. In the growth process of Pinus massoniana, our algorithms were unable to handle certain lateral stems. Moreover, if there are multiple plants in the platform at the same time and the leaves of the plants overlap, the accuracy of the algorithm will also be affected. Due to the imaging technique itself, the problem of overlapping plants and the distance between the plants and the camera is far, the information of plants collected by the imaging technique will be lacked. In this regard, we may select cameras with higher imaging accuracy, optimize the algorithms to solve the problem of overlapping stems, or use segmentation methods. This set of proposed algorithms can be applied to plant seedlings with similar structures to Pinus massoniana, such as black pine, cedar, and Pinus quinquefolius. It was proven that these algorithms can be used for these plant seedlings with some feasibility. However, every structure of plant seedlings still has slight differences, and it is also necessary to optimize the internal parameters of the algorithm and carry out more tests to improve the approaches for each plant seedling.

## Data availability statement

The raw data supporting the conclusions of this article will be made available by the authors, without undue reservation.

## Author contributions

HZ proposed and developed the approach, and HZ and YZ wrote the methods section of this article. YZ wrote other parts of the article and revised the MS. WL evaluated the accuracy of the approach. HZ designed the study. BW and ZZ improved some details of the approach. HZ acquired the 3D digitizing data. All authors contributed to the article and approved the submitted version.
