# Supplementary material for: A fast phenotype approach of 3D point clouds of Pinus massoniana seedlings
Source: Front Plant Sci. 2023 Jun 26;14:1146490. doi: 10.3389/fpls.2023.1146490 (PMC10332475; doi:10.3389/fpls.2023.1146490)
Supplement: Supplementary file 1 [file DataSheet_1.docx]

As an important step, stem and leaf segmentation could provide great convenience for the extraction of phenotypic parameters of subsequent plants. However, the main stem of Pinus massoniana seedlings was not straight, and some traditional methods, such as cylindrical fitting, were not applicable. Therefore, we propose a skeletonization method to analyze the main stem of Pinus massoniana. Regarding skeletonization, we selected some representative articles and applied the corresponding methods to Pinus massoniana seedlings for the skeletonization step. We also carried out three kinds of skeletonization methods to analyze Pinus massoniana seedlings.

The first skeletonization method was skeleton contraction. As a skeleton contraction operator, the Laplacian operator has some applications in the process of stem and leaf segmentation.([Cao et al., 2010](#_ENREF_2)) introduced the Laplace operator, the establishment process of Laplace operator is roughly as follows: The domain information of each point needed to be established. We used KDtree radius search to find the domain points of each point and projected these domain points onto the XOY plane. All points on the projection plane were processed by Delaunay triangulation. The cotangent weight of the triangle diagonal relative to each point is calculated and the Laplace operator was obtained. ([Wu et al., 2019](#_ENREF_6)) used Laplace operator to realize skeletonization and we tried this method. However, the skeletonization result was not sufficiently good, which was shown in [**FIGURE 1.A**](#Fig10). The reason was that the leaf part of Pinus massoniana seedlings was relatively concentrated, and the direction of shrinkage could not be determined. The core idea of Laplace skeletonization was mainly to search the plant point cloud in the stem direction through matrix transformation, which had a good effect on gramineous plants, such as sorghum.

The second skeletonization method was based on local feature. First, slicing and clustering were performed to extract skeleton points Some common features such as color-based feature([Tang, 2010](#_ENREF_4)), curvature-based feature([Besl et al., 1988](#_ENREF_1)),  Locally Convex Connected Patches feature([Wang et al., 2020](#_ENREF_5)) were used to determine whether the skeleton point belongs to the stem part or the leaf part. However, the leaf part and stem part of Pinus massoniana had a certain overlap, and there was no useful feature that could distinguish the stem and leaf parts.

The third type of skeletonization method was based on slicing. In ([Xiang et al., 2019](#_ENREF_7)), the Z-axis (vertical direction) was sliced, and the Euclidean clustering was used for each slice layer, and then the centroid of the clustering was extracted as the skeleton point. Then, the minimum spanning tree was used to establish the connection between skeleton points. The skeleton points were divided into three types by the connection relationship of the minimum spanning tree. The first type was leaf tip node (only one connected skeleton point), the second type was ordinary connection point (two connected skeleton points), and the third type was stem-leaf connection point (three or more connected skeleton points). Because the branches would affect the subsequent Hough plane generation effect. For the third type of nodes, pruning operations was performed. Last, a Hough plane of the skeleton points was extracted. The distances between all points and the Hough plane([Dalitz et al., 2017](#_ENREF_3)) were calculated, and the point whose distance was within the threshold was regarded as the skeleton point of the main stem. We concluded that the extraction of the Hough plane was only applicable to plants with straight stems. However, the main stem of Pinus massoniana have its own profile, therefore, some of the skeleton points of the main stem were not in the threshold range of the extracted Hough plane, resulting in some of the extracted main stem skeleton points being missed. [**FIGURE 1.B**](#Fig10) to [**FIGURE 1.D**](#Fig10) showed the process of Pinus massoniana. We also chose a plant with straight stem to compare, and the result was shown in [**FIGURE 1.E**](#Fig10) to [**FIGURE 1.G**](#Fig10). [**FIGURE 1.H**](#Fig10) showed the extraction of Hough planes and [**FIGURE 1.I**](#Fig10) showed the diagram of using Hough plane to remove the skeleton points of the main stem.

However, this method provided much inspiration for slicing skeletonization. For Pinus massoniana, the Z-axis slice layer contained both the stem part point cloud and the leaf part point cloud. Then, slice subdivision was performed along the X-axis. We compared the feature of each slice layer and found the slice layer where the main stem was located. After a deep exploration of this idea, some progress and achievements were made.

| 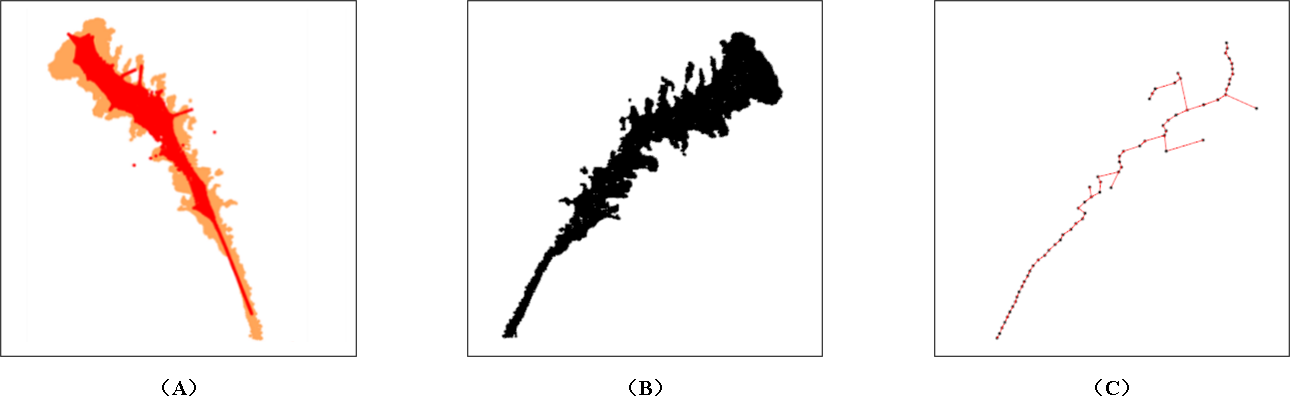  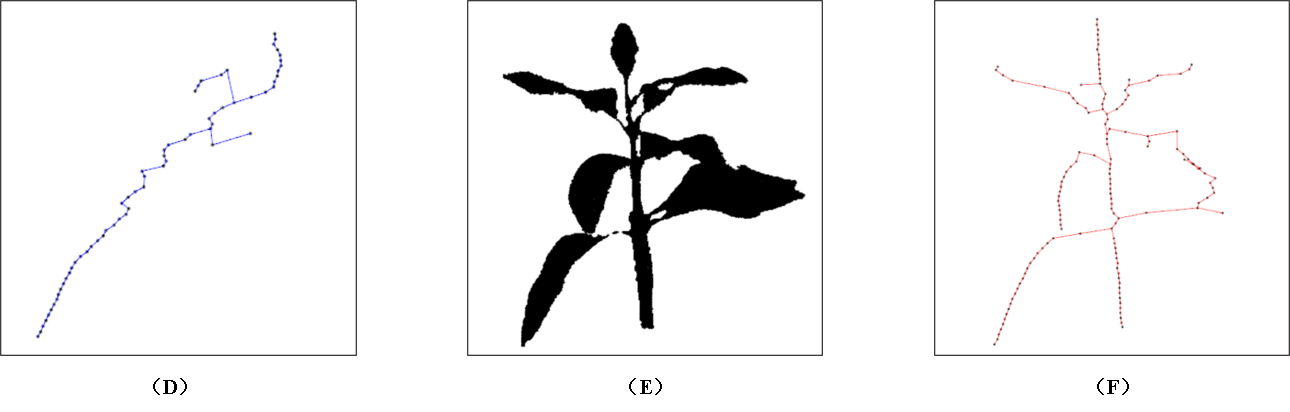  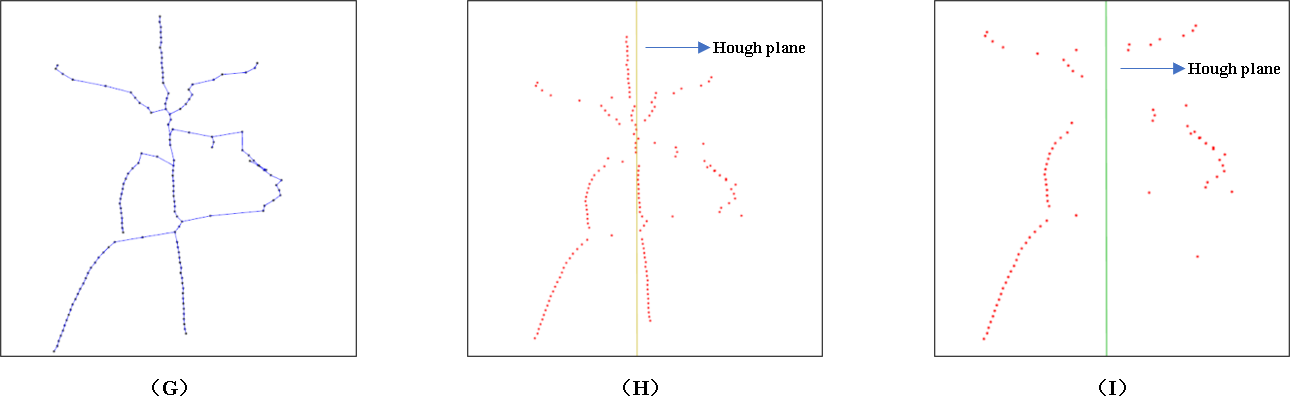  **SUPPLEMENTARY FIGURE 1**\| (A) Laplace skeletonization for Pinus massoniana seedlings. (B) Real image of Pinus massoniana. (C) Skeleton point cloud slices along the Z-axis. (D) Skeleton point cloud after pruning, here we removed branches with less than 2 skeleton points. (E) Real image of straight stem plant. (F) Skeleton point cloud slices along the Z-axis. (G) Skeleton point cloud after pruning, here we removed branches with less than 2 skeleton points. (H) The image of Hough plane. (I) Skeleton point cloud after removing main stem skeleton points. |
| --- |

Besl, P. J., Jain, R. C. J. I. T. o. p. a., & intelligence, m. (1988). Segmentation through variable-order surface fitting. IEEE Transactions on pattern analysis and machine intelligence, 10(2), 167-192. doi: [10.1109/34.3881](https://doi.org/10.1109/34.3881)

Cao, J., Tagliasacchi, A., Olson, M., Zhang, H., & Su, Z. (2010). Point cloud skeletons via laplacian based contraction. 2010 Shape Modeling International Conference, 187-197. doi: [10.1109/SMI.2010.25](https://doi.org/10.1109/SMI.2010.25)

Dalitz, C., Schramke, T., & Jeltsch, M. J. I. P. O. L. (2017). Iterative Hough transform for line detection in 3D point clouds. Image Processing On Line, 7, 184-196. doi: [10.5201/ipol.2017.208](https://doi.org/10.5201/ipol.2017.208)

Tang, J. (2010). A color image segmentation algorithm based on region growing. 2010 2nd international conference on computer engineering and technology, 6, V6-634-V636-637. doi: [10.1109/ICCET.2010.5486012](https://doi.org/10.1109/ICCET.2010.5486012)

Wang, Y., & Chen, Y. J. P. (2020). Non-destructive measurement of three-dimensional plants based on point cloud. Plants, 9(5), 571. doi: [10.3390/plants9050571](https://doi.org/10.3390/plants9050571)

Wu, S., Wen, W., Xiao, B., Guo, X., Du, J., Wang, C., & Wang, Y. J. F. i. p. s. (2019). An accurate skeleton extraction approach from 3D point clouds of maize plants. Frontiers in plant science, 10, 248. doi: [10.3389/fpls.2019.00248](https://doi.org/10.3389/fpls.2019.00248)

Xiang, L., Bao, Y., Tang, L., Ortiz, D., Salas-Fernandez, M. G. J. C., & Agriculture, E. i. (2019). Automated morphological traits extraction for sorghum plants via 3D point cloud data analysis. Computers and Electronics in Agriculture, 162, 951-961. doi: [10.1016/j.compag.2019.05.043](https://doi.org/10.1016/j.compag.2019.05.043)
